# Guidelines for surfactant selection to treat petroleum hydrocarbon-contaminated soils

**DOI:** 10.1007/s11356-021-15876-1

**Published:** 2021-09-03

**Authors:** Emilio Ritoré, Bruno Coquelet, Carmen Arnaiz, José Morillo, José Usero

**Affiliations:** 1 Departamento de Ingeniería Química y Ambiental, Escuela Técnica Superior de Ingeniería, Universidad de Sevilla, Camino de los Descubrimientos, s/n, 41092 Sevilla, Spain; 2 Inerco, Inspección y control S.A., La Cartuja Science and Technology Park, Calle Tomás Alva Edison, 2, 41092 Sevilla, Spain

**Keywords:** Soil remediation, Petroleum-contaminated soil, Surfactant, Soil organic matter, Soil texture, Aliphatic hydrocarbon, Aromatic hydrocarbon

## Abstract

The present study determined the most effective surfactants to remediate gasoline and diesel-contaminated soil integrating information from soil texture and soil organic matter. Different ranges for aliphatic and aromatic hydrocarbons (> C6–C8, > C8–C10, > C10–C12, > C12–C16, > C16–C21, and > C21–C35) in gasoline and diesel fuel were analyzed. This type of analysis has been investigated infrequently. Three types of soils (silty clay, silt loam, and loamy sand) and four surfactants (non-ionic: Brij 35 and Tween 80; anionic: SDBS and SDS) were used. The results indicated that the largest hydrocarbon desorption was 56% for silty clay soil (SDS), 59% for silt loam soil (SDBS), and 69% for loamy sand soil (SDS). Soils with large amounts of small particles showed the worst desorption efficiencies. Anionic surfactants removed more hydrocarbons than non-ionic surfactants. It was notable that preferential desorption on different hydrocarbon ranges was observed since aliphatic hydrocarbons and large ranges were the most recalcitrant compounds of gasoline and diesel fuel components. Unlike soil texture, natural organic matter concentration caused minor changes in the hydrocarbon removal rates. Based on these results, this study might be useful as a tool to select the most cost-effective surfactant knowing the soil texture and the size and chemical structure of the hydrocarbons present in a contaminated site.

## Introduction

Petroleum hydrocarbons, such as gasoline and diesel fuel, are massively used for transport and industry, causing accidental spills into groundwater and soil (Chattopadhyay and Karthick 2017; Dhaka and Chattopadhyay 2021). Anthropogenic organic compounds pollute a great number of soil environments with negative effects on human health and ecosystems (Karthick et al. 2019a). United States Environmental Protection Agency USEPA (2013) has shown that petroleum hydrocarbons are present in 70% of polluted soils in the USA. In addition, in Europe, hydrocarbons are a common pollutants, and 15% of contaminated places are caused by tank storage leaks in service stations (Panagos et al. 2013). Gasoline and diesel fuel are complex mixtures of hundreds of hydrocarbons which are clustered into two groups according to chemical structure: aliphatic and aromatic. Some of these hydrocarbons are toxic when exposed to humans, and they can cause cancer and damage to the central nervous system (Agency for Toxic Substances and Disease Registry (ATSDR) 1999).

Surfactant soil flushing is a time-efficient and versatile in situ remediation technology (Karthick et al. 2019b; Mao et al. 2015). Surfactant soil flushing depollutes the soil by making use of a solution that increases the mobility and solubility of petroleum hydrocarbons (Chattopadhyay and Karthick 2017). Surfactants are delivered into the subsoil through the aqueous phase using an infiltration or injection process to wash the hydrophobic organic compounds from soil and move them to the surfactant solution. Then, the contaminated groundwater and surfactant solution that contain contaminants are pumped to the surface through pumping wells.

Previous studies indicated that the washing of soil with non-ionic surfactants was effective for treating polluted soils. López et al. (2004) and Zhu et al. (2005) observed that the total petroleum hydrocarbon (TPH) removal rate by non-ionic surfactants was up to 60%. In addition, Baziar et al. (2013) obtained the best removal efficiencies with Tween 80 and Brij 35 at 80 and 65%, respectively. On the other hand, the results of Chevalier (2003) and Deshpande et al. (1999) suggest that anionic surfactants are suitable for remediating petroleum hydrocarbon-contaminated sites. Also, Khalladi et al. (2009) removed 97% of diesel fuel with SDS in a column laboratory study. Mineral surfaces and soil organic matter are mostly negatively charged. Cationic surfactants are unsuitable for surfactant soil flushing, due to the fact that they tend to adsorb onto the negatively charged surfaces of soil by electrostatic forces (Paria and Yuet 2006). This interaction causes surfactant loss and demand higher concentrations in the solution for micelle creation (Ishiguro and Koopal 2016). For this reason, the best surfactants for hydrocarbon removal seem to be the anionic and non-ionic.

Several soil components adsorb surfactant monomers because of their properties; thus, sorption of surfactant–soil is an important parameter for surfactant soil flushing. Surfactant efficiency for solubilizing petroleum hydrocarbons decreases when the soil adsorbs a significant amount of it. The surfactant–soil sorption depends on soil texture (Karthick et al. 2019c; Paria 2008), soil organic matter (Ussawarujikulchai 2008) and the type of surfactant used (Paria 2008). Soil texture and soil organic matter influence in surfactant soil flushing has been studied in polycyclic aromatic hydrocarbons (PAHs) (Ussawarujikulchai 2008; Zhou and Zhu 2007) and chlorinated organic compounds (Lee et al. 2002). Nevertheless, little is known about gasoline and diesel (Yan et al. 2016). In addition, previous works about petroleum hydrocarbons analyzed the efficacy of surfactants only for total petroleum hydrocarbon (TPH) removal (Baziar et al. 2013; Chevalier 2003; Deshpande et al. 1999; Karthick and Chattopadhyay 2017; López et al. 2004; Vreysen and Maes 2005; Zhu et al. 2005) or for representative compounds such as toluene, decane, or dodecane (Atteia et al. 2017; Jousse et al. 2017; Pennell et al. 1993). Surfactant selection is an important aspect to consider because each soil flushing process is different since it depends on the soil and the contaminants as no two contaminant–soil combinations are the same.

The objective of the present work is to present novel guidelines in order to determine the best surfactants for remediating gasoline and diesel-contaminated soil according to soil texture and soil organic matter. Little is known about the effects of soil organic matter and soil texture in a gasoline- and diesel-polluted soil. In addition, not only TPH were analyzed but also the need to study aromatic and aliphatic hydrocarbons as well as different fraction ranges. Hydrocarbon fractions and surfactant desorption have been scarcely investigated; few authors have studied the preferential desorption of different hydrocarbon fractions during soil washing with surfactants. Besides TPH analysis, the effect of surfactant on the most widely represented components found in gasoline and diesel was determined during each laboratory test, in order to elucidate whether there is a preferential desorption of petroleum hydrocarbon compounds. It is essential to investigate the desorption rates of different types of hydrocarbons because gasoline and diesel fuel contain compounds that are both easily removable and recalcitrant.

## Materials and methods

### Materials

Three natural soils from Andalusia, south of Spain (Los Marines, Almonte, and La Puebla del Río) were collected from uncontaminated sites at the depth of 10–40 cm. The soil samples were selected to obtain a wide range in sand, silt, and clay contents.

A detailed comparison of the removal of hydrocarbon fractions that appear in gasoline and diesel fuel was carried out with various surfactants in different soils. Brij 35 (purity > 99%), Tween 80 (purity > 99%), and sodium dodecylbenzenesulfonate (SDBS, purity > 99%) were supplied by Sigma-Aldrich, and sodium dodecyl sulfate (SDS, purity > 99%) was obtained from Panreac Applichem. They were selected for this study because of their low toxicity (Cheng et al. 2018; The Soap and Detergent Association (SDA) 1994, 1991), high potential for biodegradation (Cheng et al. 2018; Federle and Itrich 2006; Gustav et al. 2011; Tabor and Barber 1996), great solubilization capacity, and high volume of production in industry. The characteristics of the surfactants are listed in Table 1. The minimum surfactant concentration at which micelles begin to form is called the “critical micelle concentration (CMC)” and at supra-CMC surfactant inclusion participates to the formation of additional micelles. Surfactant solutions were prepared by dissolving surfactants in Milli-Q water type II water.

**Table 1:** Properties of surfactants

Commercial surfactant name	Chemical name	Type	Molecular weight (g mol^−1^)	CMC (mM)
Brij 35	Polyoxyethylene lauryl ether	Non-ionic	1200	0.09 (Baziar et al. 2013)
SDBS	Sodium dodecyl benzenesulfonate	Anionic	348	2.76 (Zhao et al. 2005)
SDS	Sodium dodecyl sulfate	Anionic	288	8.2 (Ceschia et al. 2014)
Tween 80	Polyoxyethylene (20) sorbitanmonooleate	Non-ionic	1310	0.01 (Tsai and Kao 2009)

*CMC* critical micelle concentration

The gasoline and diesel fuel used in this work were commercially available and they were obtained from a petrol station.

### Soil preparation

Prior to using the soil, it was homogeneously mixed and air dried. The samples were tapped to break aggregated soil and passed through a 0.5-mm sieve. Then, the soil was artificially contaminated by slowly adding a mixture gasoline and diesel fuel (60–40%) with continuous mixing, due to the fact that in many hydrocarbon-contaminated places, such as fuel stations, it is usual to find a mixture of these fuels as pollutants. The polluted soil was kept out of light in a closed vessel for 14 days. Then, the initial concentration of soil contaminant hydrocarbons was analyzed by the gas chromatography/mass spectrometry (GC-MS) method.

For elucidating the role of soil organic matter, Los Marines soil was used. Different organic matter contents were required. The soil with lower amount of organic matter (0%) was obtained after calcination of the collected soil for 24 h at 550 °C in a muffle furnace (Nabertherm, 19/12/S27). The soil with the highest content of organic matter was Los Marines soil without heat treatment (5%). Finally, a soil with an intermediate amount of organic matter was made by a mixture of untreated soil and calcined soil in a 1:1 ratio; in this way, a soil with an organic matter of 2.5% was obtained.

### Laboratory soil flushing experiments

Samples of 200 g of the spiked soil were placed in 2-L glass bottles with screw Teflon® caps. The glass bottles were kept out of light at 18–20 °C for 14 days. Afterwards, 1.6 L of surfactant solution (soil/water ratio of 1:8) were added at a concentration of 1.5%. The surfactant concentration and soil/water ratio have been chosen according to previous studies. Baziar et al. (2013) showed that a concentration around 1.5% is the optimal surfactant concentration for the removal of hydrocarbons. Higher surfactant concentrations did not show a significant hydrocarbon removal. A 1:8 soil/water ratio has been chosen because Peng et al. (2011) indicated that the optimal ratio was between 1:8 and 1:10 soil/water ratios for surfactant washing. Desorption experiments were performed with different types of surfactants. The soil–solution mixtures in the capped glass bottles were shaken twice and then left to rest for 24 h in the dark. To separate the aqueous and solid phases in bench experiments, the samples were centrifuged at 3500 rpm for 15 min. Milli-Q type II water as washing solution was also prepared as a control experiment. Ten grams of the soil was analyzed for hydrocarbons. The amount of hydrocarbons removed was computed from the difference of the initial and final concentrations. The removal rate of gasoline and diesel fuel hydrocarbons from soil was determined from Eq. 1:

1 $$ \mathrm{Removalrate}\left(\%\right)=\left({\mathrm{C}}_{\mathrm{i}}-{\mathrm{C}}_{\mathrm{f}}\right)/{\mathrm{C}}_{\mathrm{f}}\times 100 $$

where *C*_*i*_ (mg/kg) is the initial hydrocarbon soil concentration and *C*_*f*_ (mg/kg) is the final hydrocarbon concentration after water or surfactant solution washing.

### Analysis methods

All analyses were performed in triplicate. Soil textures were determined by the pipette method (The Royal Netherlands Standardization Institute (NEN) 2018). The amount of organic carbon in the soil was determined by gravimetric analysis (The Royal Netherlands Standardization Institute (NEN) 1992) and the pH of the soil was measured with a pH meter Thermo 920A following a procedure similar to the one reported by Fernández Linares et al. (2006).

The volatile hydrocarbon group (> C6–C10) was determined by gas chromatography/mass spectrometry (GC/MS) according to USEPA 8260b (United States Environmental Protection Agency USEPA 1996a) and USEPA 5021a (United States Environmental Protection Agency USEPA 2014). The head space method consisted of heating at 80 °C for 1 h 10 g of soil in a glass vial. Then, the gas phase was injected into a GC. We used an Agilent Technologies 6890N with a capillary column (3 m × 0.54 mm × 0.85 μm). Injector and detector temperature was 250 °C and it was programmed to increase from 70 to 115 °C at 5 °C min^−1^. The carrier gas was helium and the flow rate was 3.9 mL min^−1^. Volatile hydrocarbons analysis was run in 8.2 min. External standards were used.

A gas chromatography equipped with a flame ionization detector (GC/FID) was used for the detection of non-volatile hydrocarbons (> C10–C35) (United States Environmental Protection Agency USEPA 1996b). The following capillary column were used: 30 m × 0.25 mm × 0.25 μm. The injector and detector temperature was 280 °C. The furnace temperature was increased from 45 to 250 °C at 12 °C min^−1^ and held at this temperature for 15 min. Helium was the carrier gas at a flow rate of 10 mL min^−1^. External multilevel calibrations were carried out for oil fractions. Previously, the samples of hydrocarbons were introduced into the GC according to the solvent extraction method, Soxhlet extraction (United States Environmental Protection Agency USEPA 1996c, 1994).

### Statistical analysis

Data obtained from the laboratory experiments were analyzed by SPSS (version 21) and statistical significance was determined by either the *t* test or analysis of variance (ANOVA) and Tukey test.

## Results and discussion

### Soil characteristics

The total hydrocarbon (C6–C35) content in the soil was checked to be 8530 mg/kg (Table 2). Table 2 provides the data about the hydrocarbon concentration for different fractions in soil after the contamination in the laboratory. The results show a high level of gasoline and diesel fuel hydrocarbon range. The properties of the soil samples are listed in Table 3. The soils present heterogeneous characteristics.

**Table 2:** Initial hydrocarbon concentration (mg hydrocarbon/kg soil)

> C6–C8	> C8–C10	> C10–C12	> C12–C16	> C16–C21	> C21–C35	Total
Aliphatic hydrocarbon concentration (mg/kg soil)						
820	610	580	1300	1410	640	5360
Aromatic hydrocarbon concentration (mg/kg soil)						
690	870	570	390	510	140	3170
						8530

**Table 3:** Soil properties

Soil	Properties	Value		
La Puebla del Río	Particle size distribution (%)	Sand	Silt	Clay
		3.8	53.5	42.7
	Texture	Silty clay		
	Organic matter (%)	7.2		
	pH	8.3		
Los Marines	Particle size distribution (%)	Sand	Silt	Clay
		20.6	58.2	21.2
	Texture	Silt loam		
	Organic matter (%)	5.1		
	pH	5.9		
Almonte	Particle size distribution (%)	Sand	Silt	Clay
		86.2	12.4	1.4
	Texture	Loamy sand		
	Organic matter (%)	1.5		
	pH	6.5		

### Effect of soil texture on washing surfactant

**Fig. 1:**
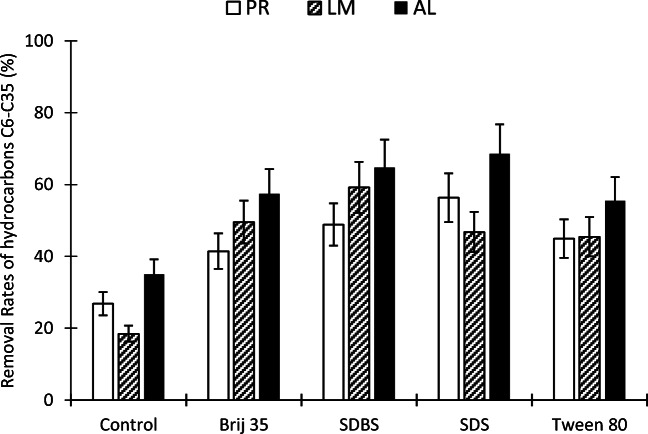
Desorption efficiencies of surfactants for petroleum hydrocarbons adsorbed by different soils. PR = La Puebla del Río (silty clay), LM = Los Marines (silt loam), AL = Almonte (loamy sand)

Petroleum hydrocarbons in the soil were analyzed after washing three Andalusian soils using four surfactants. Figure 1 shows the removal rates of total petroleum hydrocarbons (> C6–C35) achieved for batch experiments. La Puebla del Río, Los Marines, and Almonte polluted soils were washed with the selected surfactants. The results indicated that hydrocarbon removal was limited in the control sample (water as washing solution). Figure 1 illustrates that the water effect (control) was lower than 40% for all of the soils: silty clay (27%), silt loam (18%), and loamy sand (35%). These results are consistent with Khalladi et al. (2009), who obtained similar petroleum hydrocarbon desorption rates using water in column experiments.

The sequence in terms of the removal efficiency of petroleum hydrocarbons was as follows: La Puebla del Río (silty clay) < Los Marines (silt loam) < Almonte (loamy sand). All surfactants removed more contaminants from the Almonte soil than from other soils. The surfactant removal rates from loamy sand soil followed the following order: SDS > SBDS > Brij 35 > Tween 80 (Fig. 1). The efficiency of all surfactants in Almonte soil was above 60%, and the highest was SDS (69%).

In the silt loam-type soil, Los Marines, the highest efficiency among the surfactants was 59% (SDBS) and the lowest efficiency was 45% (Tween 80), while the surfactants SDS and Brij 35 obtained analogous results, at 47 and 50%, respectively (Fig. 1).

Lastly, the surfactant efficiency for removing petroleum hydrocarbons from the loamy sand-type soil, La Puebla del Rio, was the lowest compared to the other two soils and followed the following order: SDS > SDBS > Tween 80 > Brij 35 (Fig. 1). Brij 35 was the least efficient surfactant and achieved a hydrophobic organic compound desorption rate of 41%, which is slightly better than the control (27%). The best surfactant for La Puebla del Rio soil samples was SDS, which removed 56% of hydrocarbons. SDBS and Tween 80 surfactants reached intermediate desorption, at 49 and 45%, respectively.

The results shown above indicate that soil texture must be considered in surfactant soil flushing projects based on the different hydrocarbon desorption rates observed in laboratory experiments. Figure 1 suggests that as the size of the soil particles decreases (the silt and clay content increases), the desorption of the total hydrocarbons (> C6–C35) by means of the washing with the four studied surfactants drops. These results could be described mainly for two reasons: a greater capillary force in soils with fine particles and the soil–surfactant adsorption processes. The adsorption of surfactants depends basically on the type of particles in the soil and on the characteristics of the surfactant.

These results are not in line with those reported by Jousse et al. (2017) because they suggested that soil texture is not an important parameter. They resolved that grain size of soil do not show clear effects when using Tween 80 for remediating a hydrophobic organic compound-polluted soil. Notwithstanding, our data shown in Fig. 1 agree with other previous literature such as Lee et al. (2002), who found that the removal of hydrophobic aromatic compounds decreases with high clay contents in soil. Other authors such as Brownawell et al. (1997), Ou et al. (1996), Podoll et al. (1987), Shen (2000), and Yang et al. (2007) demonstrated that surfactant monomers are adsorbed by the clay’s surface. These authors have used the same type of surfactant as us, anionic and non-ionic. Ou et al. (1996) and Yang et al. (2007) used SDBS (the same surfactant we used) and Brownawell et al. (1997) and Podoll et al. (1987) used surfactants from the same family as Brij 35, alcohol ethoxylates. The surfactant adsorption is positively related to the clay content in the soil. Rodríguez-Cruz et al. (2005) obtained a 79% correlation between the amount of clay and the Triton X-100 (non-ionic surfactant) adsorption. Clay mineralogy plays an important role in surfactant adsorption. Clay with high silica content such as bentonite can adsorb more surfactants than soils with large amounts of iron such as red soils (Shen 2000). For this reason, clay soils rich in clays with 2:1 silicate structure are not recommended to carry out a successful surfactant treatment due to higher surfactant sorption. On the other hand 1:1 clays such as kaolinite and gibbsite have lower sorption; a 2:1 structure has a greater amount of Si/Al than 1:1 clays (Zhu et al. 2003). Shen (2000) proved that greater non-ionic surfactant sorption ability was exhibited by soil with a larger Si/(Al + Fe) ratio. Furthermore, small-sized grains increase capillaries, resulting in a stronger bond between soil particles and oil (Lake 1998). The higher surface area of the small-sized grain increases the forces that trap the hydrocarbons into soil pores. This study suggests that sandy soils are more available for surfactant soil flushing than soils with a higher amount of clay because those soil components decrease surfactant efficacy. Thus, in order to increase hydrocarbon desorption from clay soils, a greater amount of surfactant must be used. Surfactant soil flushing could be rejected as a remediation method if large amounts of surfactants are necessary to achieve high hydrocarbon desorption rates.

With respect to the comparison of the surfactants tested, the data show that Brij 35 and Tween 80 significantly increased hydrocarbon desorption in relation to the control experiment (Fig. 1). In Almonte soil, they desorbed 62 and 57%, respectively, more than in the control test, in Los Marines soil 177 and 150%, respectively, and in La Puebla del Río soil 52 and 66%, respectively. These results show that the surfactant treatment notably increases petroleum hydrocarbon desorption, especially in silt loam soil with respect to water washing. Nonetheless, it was notable that anionic surfactants (SDS and SDBS) removed more petroleum hydrocarbons than non-ionic surfactants (Brij 35 and Tween 80). SDBS and SDS attained the best results for loamy sand soil (65 and 69% of removal rates, respectively) and in silty clay soil (49 and 56%, respectively). However, anionic surfactants achieved the first (SDBS, 59%) and the third (SDS, 47%) best results in silt loam soil, while Brij 35 attained the second best result, with a 50% desorption rate. The considerably high concentration of CMC used during these experiments increased the desorption of organic pollutants through the mobilization and dissolution mechanisms. The 1.5% of surfactant concentration used in terms of CMC varied among the surfactants. 1.5% of Brij 35, SDBS, SDS, and Tween 80 correspond to 139 CMC, 16 CMC, 6 CMC, and 1145 CMC, respectively. These data indicate that non-ionic surfactants should achieve better hydrocarbon removals than anionic ones because in terms of CMC, they show a concentration higher with the same amount of surfactant (1.5%_m/v_). However, the results show that anionic surfactants eliminate more hydrocarbons than non-ionic surfactants. The dissimilar removal rates observed between anionic and non-ionic surfactants could be explained by surfactant–soil sorption. This result supports the works of Muherei et al. (2009) and Rodríguez-Cruz et al. (2005), who observed less adsorption affinities of different soils to SDS compared to TX100 (non-ionic surfactant). They suggested that repulsive electrostatic interactions appear between anionic surfactant and soil surfaces because the majority are negatively charged, causing higher adsorption in the soil for non-ionic surfactants than anionic surfactants. In the soil/aqueous system, the solubilization of hydrocarbons occurs at surfactant dosages greater than the water surfactant CMC (Liu et al. 1992). The larger surfactant concentration at which solubilization starts in the presence of soil can be called “effective CMC” (Zheng and Obbard 2002). A high adsorption of surfactant monomers by the soil reduces the amount of micelles that can be formed, decreasing the removal of pollutants. For this reason, despite the high concentrations in terms of CMC of the non-ionic surfactant used, they did not show a higher performance than anionic surfactants.

### Desorption of different hydrocarbon compounds in different soils

**Fig. 2:**
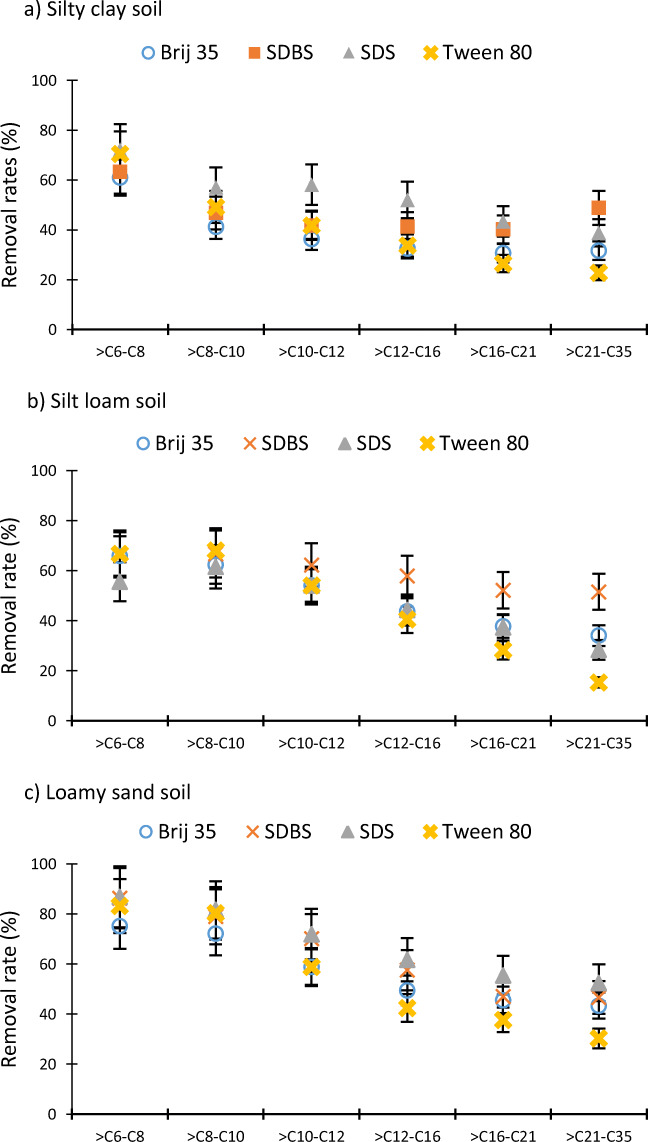
Desorption rates for hydrocarbon ranges in the three tested soils. **a** La Puebla del Rio soil, **b** Los Marines soil, and **c** Almonte soil

**Fig. 3:**
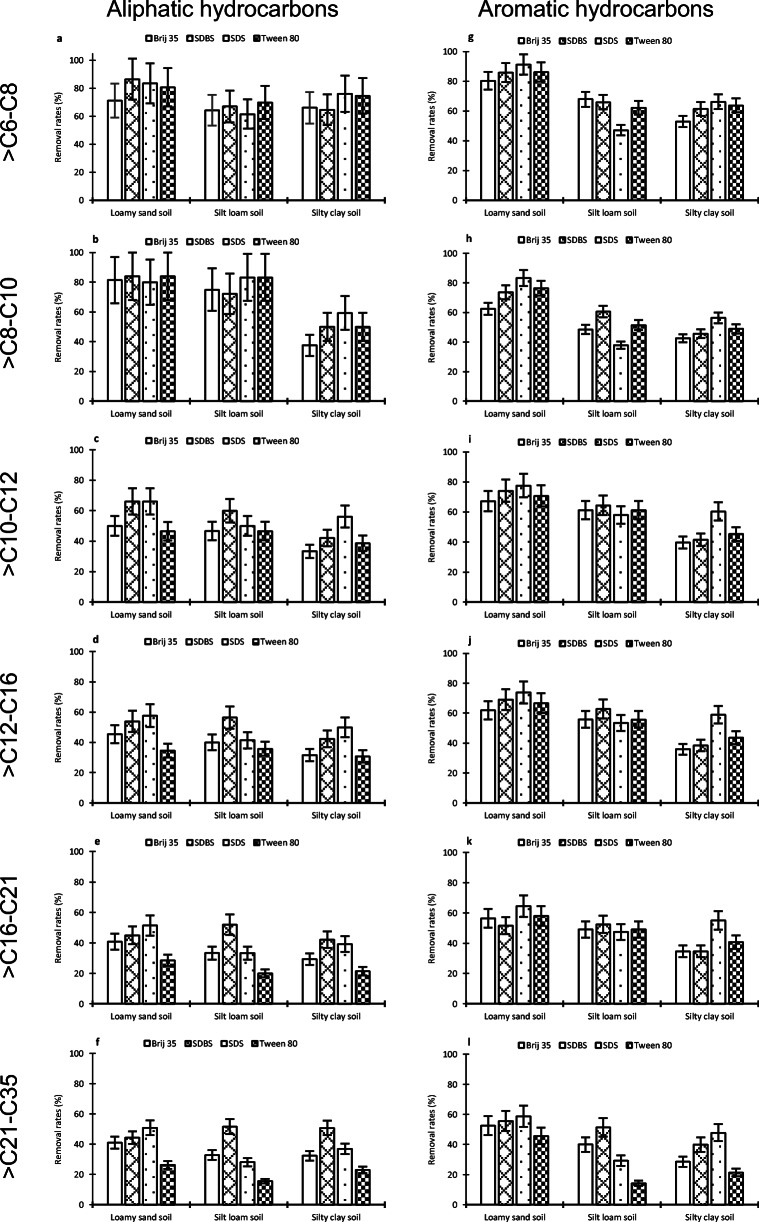
**a**–**l** Desorption efficiencies for different hydrocarbon fractions

Figures 2 and 3 show the desorption of different pollutants on the three soils tested. In addition, desorption data are grouped into hydrocarbon ranges and into aliphatic and aromatic organic compounds.

Figure 2 indicates the hydrocarbon fraction desorption percentage for the three tested soils (Fig. 2a La Puebla del Rio soil, Fig. 2b Los Marines soil, and Fig. 2c Almonte soil). The results shown in Fig. 2 suggest that desorption decreases when the size of hydrocarbon compounds rises because, in all samples, the removal rates are lower for higher-sized hydrocarbon fractions compared to smaller ranges.

Hydrocarbon fraction desorption in the silty clay soil (Fig. 2a) showed a continuous decrease for surfactants SDS and Tween 80 at the same time that the hydrocarbon size increased. SDS and Tween 80 achieved 72 and 70% of > C6–C8 hydrocarbon desorption, respectively, but their effectiveness was reduced, only removing 39 and 23% of hydrocarbons with between 22 and 35 carbons. However, the hydrocarbon removal in this soil for the rest of the surfactants in ranges greater than > C10–C12 was stable, as approximately the same percentages of these hydrocarbon fractions were desorbed. Brij 35 and SDBS managed around 35 and 45% removal rates for these hydrocarbon fractions (Fig. 2a). In addition, for the ranges > C12–C16, > C16–C21, and > C21–C35 in silty clay soil, the anionic surfactants obtained better desorption rates than non-ionic ones. SDBS and SDS removed 41 and 52% of fraction > C12–C16, 40 and 43% of fraction > C16–C21, and 49 and 39% of fraction > C21–C35, respectively, while Brij 35 and Tween 80 desorbed 33 and 34% of fraction > C12–C16, 31 and 26% of fraction > C16–C21, and 32 and 23% of fraction > C21–C35, respectively (Fig. 2a).

In the silt loam soil, the SDBS surfactant showed a peculiarity in respect to the rest of the surfactants. In the manner that SDBS removal efficiency on the organic compounds from gasoline and diesel is not reduced as the hydrocarbon fraction increases. This could be related to micelle sizes of the surfactants used in this study. The micelle core radius reported in the literature are 1.7 nm for Brij 35 (Preu et al. 1999), 2 nm for SDBS (Palazzesi et al. 2011), 1.75 nm for SDS (Duplâtre et al. 1996), and 1.42 nm for Tween 80 (Karjiban et al. 2012). A larger micelle core radius can solubilize the highest petroleum hydrocarbons more effectively. The surfactant SDBS forms the largest micelles; this may elucidate why SDBS has not reduced the removal efficiency as the hydrocarbon range rises. Thus, SDBS does not present preferential desorption based on the size of the hydrocarbons on silt loam soil samples and always shows a desorption rate around 60% of elimination over the soil for all the analyzed fractions (Fig. 2b). In this case, the results suggest that SDBS could be a suitable surfactant for hydrophobic organic compound-polluted soils that contain very different hydrocarbons, such as gasoline and diesel, because it achieves similar efficacy on all compounds regardless of the size of the compounds. SDBS is the surfactant with the least similarity between hydrocarbon size and desorption rate. Nonetheless, in the other three surfactants in silt loam soil, whenever the hydrocarbon size increases, their desorption efficiencies are reduced; Brij 35, SDS, and Tween 80 attained 66, 56, and 67%, respectively, of > C6–C8 hydrocarbon removal rates, whereas the largest hydrocarbons (> C21–C35) were desorbed by 34, 28, and 15% by Brij 35, SDS, and Tween 80, respectively (Fig. 2b).

The four surfactants clearly decreased the desorption rates while increasing the hydrocarbon ranges in loamy sand soil. For > C6–C8 fraction, the maximum desorption rate was 88% and the efficiency was reduced to around 50% in the > C21–C35 fraction (Fig. 2c). This results are not in line with those reported by Khalladi et al. (2009) that analyzed the SDS effect on diesel n-alkane (C8–C26) contaminated sandy soil and suggested that SDS did not sufficiently remove the n-alkanes present in diesel fuel.

The largest desorption efficiency reduction, when increasing the fraction size, was observed using Tween 80 on all soil samples (Fig. 2). In the silty clay soil, Tween 80 desorbed 70% of > C6–C8 hydrocarbons, but only 23% of C21–C35 (a difference of 53%); 67% of > C6–C8 hydrocarbons, but only 15% of C21–C35 (a difference of 52%); and 83% of > C6–C8 hydrocarbons, but only 30% of C21–C35 (a difference of 47%) (Fig. 2a). Tween 80 is the surfactant that is most sensitive to the variation in the size of hydrocarbons. It was significant that the surfactant attained a lower effectiveness in the reduction in larger compounds. These poorer desorption rates were compared to other surfactants in the largest hydrocarbon ranges (> C12–C16, > C16–C21, and > C21–C35). The results for Tween 80 are consistent with Li et al. (2016), who used Tween 20 (similar to Tween 80 but with less ethylene oxide in the tail to solubilize petroleum hydrocarbons. They reported that Tween 20 was ineffective for treating heavy petroleum hydrocarbons in different clays.

Figure 3 shows desorption rates for different hydrocarbon fractions clustering aliphatic and aromatic hydrocarbons. Figure 3 is focused on indicating the differences between aliphatic hydrocarbon soil removal compared to aromatic hydrocarbons. The aim is set on checking whether the four used surfactants in soils with textural differences exert a preferential desorption over any of the groups of compounds that are included in gasoline and diesel fuels.

On the one hand, surfactants achieved around 72% of aliphatic hydrocarbon removal for the > C6–C8 range, but desorption gradually decreased to 36% (> C21–C35) (Fig. 3a–c). On the other hand, aromatic hydrocarbons managed a significant reduction. For example, in the loamy sand, the highest elimination value was 91 and 83% for fractions > C6–C8 and > C8–C10, respectively, and the lowest less than 55% for > C21–C35 (Fig. 3c). The reduction in desorption as the hydrocarbon size increases is lower in aromatic hydrocarbons compared to aliphatic ones. This could be explained based on chemical structure of hydrocarbons. For the same range, aliphatic hydrocarbons have a higher molecular mass than aromatic ones. For example, aliphatic hydrocarbons have a molecular mass in the ranges C10–C12, C12–C16, C16–C21, and C21–C36 of 159,186, 242, and 338 g/mol, respectively. On the other hand, aromatic hydrocarbons have a molecular masses 130, 145, 173, and 216 g/mol for the same fractions (Alaska Statement of Cooperation Working Group 2006). The difference in molecular masses between aromatic and aliphatic hydrocarbons increases as their size rises. This greater gap in molecular mass in the higher fractions may explain the desorption reduction. For this reason, when the hydrocarbon size increases, the solubilization by surfactant micelles of aliphatic compounds decreases because its molecular mass increases faster as the fraction rises with respect to aromatic hydrocarbon.

The analysis of aliphatic compounds indicates that, in general, for each range of hydrocarbons, the removal efficiency is similar in the three studied soils. Nevertheless, aromatic hydrocarbons showed differences, mainly on loamy sand soil (Fig. 3). This soil attained the best desorption rates in relation to silt loam and silty clay soil. Figure 3 shows desorption rates for hydrocarbon fractions of between 19 and 28% higher.

In general, aromatic compounds were desorbed more easily than aliphatic compounds in the three tested soils. This trend is especially evident in the compounds with more than ten carbons. For instance, the SDS surfactant in loamy sand soil (Fig. 3i) attained a desorption rate of 78% for > C10–C12 aromatic hydrocarbons, while only 66% of aliphatic hydrocarbons were removed (Fig. 3c). This difference between aliphatic and aromatic hydrocarbons was observed in all the ranges studied (Fig. 3). These results are not in agreement with Urum et al. (2006). They investigated SDS efficiency on crude oil-contaminated soil and they determined that this anionic surfactant desorbed more of the aliphatic than the aromatic compounds. However, similar results to this work were recently reported by Jousse et al. (2017), who achieved better contaminant removal for toluene (aromatic hydrocarbon) than n-decane (aliphatic hydrocarbon). This could be explained by using the octanol–water partition coefficient (*K*_OW_). *K*_OW_ is a measure of the relative attraction of a compound from the solid or organic liquid phase and water (a high *K*_OW_ indicates a high preference for the nonpolar material). In this case, *K*_OW_ quantifies the relative affinity of hydrocarbon clusters to soil and surfactant solutions. Petroleum hydrocarbons are hydrophobic organic compounds with a high *K*_OW_, and for this reason, they are strongly bound to some components in the soil. Aromatic hydrocarbons have a smaller *K*_OW_ value than aliphatic ones (Table 4). The value shown in Table 4 indicates that in the comparison between the compounds with the same size (equal number of carbons), the octanol–water partition coefficient in the aromatic compounds is lower than that of the aliphatic ones. Because of this, aromatics are less sorbed in the soil and are less recalcitrant than aliphatics for surfactant remediation. In the same way, the number of carbons is positively correlated with *K*_OW_, and the results observed in this study are consistent with the relation between the octanol–water partition coefficient and hydrocarbon removal using surfactant solutions. Hydrocarbons with a lower *K*_OW_ value are more soluble in water and more available to be solubilized by the micellar phase of the surfactant solution. The results of this test confirm the research of Zhou and Zhu (2005), who suggested a model that correlates with the *K*_OW_ of several polycyclic aromatic hydrocarbons (phenanthrene, fluorine, acenaphthene, and naphthalene) and hydrocarbon soil desorption.

**Table 4:** Octanol–water partition coefficient (log *K*_OW_) (The Risk Assessment Information System 2015)

	> C6–C8	> C8–C10	> C10–C12	> C12–C16	> C16–C21	> C21–C35
Aliphatic	3.78	4.76	5.74	7.22	9.18	13.6
Aromatic	2.43	3.15	3.72	4.46	5.61	7.28

Figure 3 analyzes the initial behavior of the more abundant fractions. The main hydrocarbon fractions were as follows (Table 2): aliphatic > C16–C21 (1410 mg/kg), aliphatic > C12–C16 (1.300 mg/kg), and aromatic > C8–C10 (870 mg/kg). For the most abundant hydrocarbon fraction, > C16–C21, it can be observed that Tween 80 only managed to eliminate around 20% of hydrocarbons from all the soil samples. The other three surfactants desorbed more organic compounds from this fraction, achieving a maximum of 51% desorption rate with the SDS surfactant (Fig. 3c). In loamy sand soil, Brij 35, SDBS, and SDS attained percentages of removal higher than 40% (Fig. 3c), while in other soils, only SDBS exceeded this percentage, with desorption rates of 52 and 42% in silt loam soil and silty clay soil, respectively (Fig. 3a, b). Very similar results were observed for the aliphatic fraction > C12–C16, with only a slightly higher hydrocarbon removal since they are smaller hydrocarbons than aliphatic ones > C16–C21. The third important range in terms of abundance in the initial concentration of hydrocarbons in the treated contamination are aromatics > C8–C10. Figure 3c shows that these hydrocarbons in loamy sand soil were mostly removed, around 75% (63% Brij 35, 74% SDBS, 83% SDS, and 76% Tween 80). These removal rates were greater than the other two tested soils that achieved around 50% of removal rates for silt loam soil (48% Brij 35, 61% SDBS, 38% SDS, and 52% Tween 80) and silty clay soil (43% Brij 35, 46% SDBS, 56% SDS, and 49% Tween 80). The difference in this fraction is noticeable when compared to aliphatic hydrocarbons of the same size. In a loamy sand soil, aliphatic and aromatic hydrocarbons are removed in the same order of magnitude but, in silt loam soil, aromatic hydrocarbons reach a higher elimination rate (between 20 and 40%) depending on the type of surfactant studied. In the silty clay soil, for the aromatic fraction > C8–C10, the surfactants presented poorer results with respect to the other two soils. Only around 50% (43% Brij 35, 46% SDBS, 56% SDS, and 49% Tween 80) of the hydrocarbons that were initially present in the contaminated soil are desorbed (Fig. 3a).

### Soil organic matter influence

**Fig. 4:**
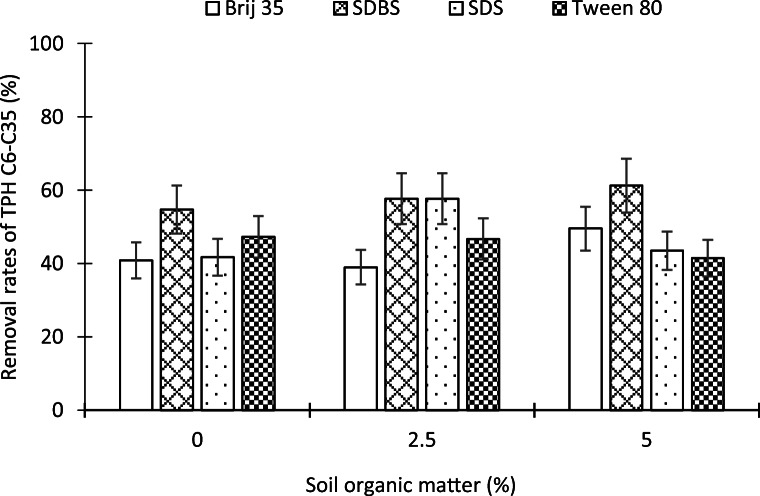
Influence of soil organic matter on the total petroleum hydrocarbons (> C6–C35) removal rate

Figure 4 shows the removal rates of petroleum hydrocarbons (C6–C35) on soil samples with three different organic matter concentrations (0%, 2.5%, and 5%). Four surfactants were tested (Brij 35, SDS, SDBS, and Tween 80) in order to analyze the soil organic matter influence on surfactant soil flushing. The results indicated that there are limited changes in the removal percentages associated with the soil organic matter variation. The hydrocarbon desorption rates were always between 40 and 60% of the initial soil hydrocarbon concentration (Fig. 4). In the soil without organic matter, 41%, 55%, 42%, and 47% of hydrophobic organic compounds are eliminated, with the surfactants Brij 35, SDBS, SDS, and Tween 80, respectively. On the other hand, in the soil with 2.5% of organic matter, 39% (Brij 35), 58% (SBDS), 58% (SDS), and 47% (Tween 80) of pollutants were desorbed. Finally, in the soil with the higher content of organic matter (5%), the surfactants Brij 35, SDBS, SDS, and Tween 80 achieved petroleum hydrocarbon removal rates of 50%, 59%, 47%, and 47%, respectively. Therefore, the concentration of soil organic matter, in the range tested (0–5%), is a factor with slight relevance on the desorption of total petroleum hydrocarbons (C6–C35) of the polluted soil by surfactant washing. The soil organic matter level in most natural soils ranges from 1 to 3.5%. Since these values are lower than the maximum concentration used in the present research work, soil organic matter could be considered as a parameter without relevance in most of the surfactant remediation process compared to other factors such as the surfactant type or the soil granulometry.

No previous research has been found exploring the effect of organic matter on the desorption of gasoline and diesel using soil samples with organic matter concentration in a natural range. Only in the case of a very high concentration (50%) of organic matter artificially added to the soil has been demonstrated, for the naphthalene hydrocarbon, that the soil desorption depends on the soil organic matter content (Ussawarujikulchai 2008). Furthermore, Yan et al. (2016) remediated two diesel-contaminated soils with a solution of the surfactant Tween 80. The soils had different soil organic matter contents (1.1 and 2.7%). A greater elimination of diesel fuel (67.5%) was achieved in the soil with a lower concentration of organic matter, and on the other hand, only 45.4% of total petroleum hydrocarbons was removed from the soil with a higher concentration of soil organic matter in the soil. Yan et al. (2016) suggest that these results could be explained by the difference in soil organic matter concentration, soil organic matter adsorb surfactants reducing their effectiveness to act on organic pollutants. The results of Yan et al. (2016) differ with our research; although in their study, the results may be due to the different granulometry of the soils. The soil with a finer grain size has shown the worst results; this soil had 70% of silt and clay while the other soil only had 40%. Previous studies (Lee et al. 2002; Li et al. 2016; Rodríguez-Cruz et al. 2005; Zhou and Zhu 2007) and this research indicated the influence of soil texture on surfactant washing of organic pollutants. The clays are a negative influence on surfactant performance because they adsorb surfactant monomers, inhibiting them from solubilizing hydrophobic organic compounds.

## Conclusions

The results provide useful information about surfactant selection based on soil texture and soil organic matter for removing hydrocarbons from polluted soils. Summarizing the main points, the hydrocarbon desorption percentage in soils indicates that soils with higher clay content show less hydrocarbon compound desorption, that is, the hydrocarbon removal efficiency decreases while the amount of small soil particles increases. The surfactant comparison notes that anionic surfactants attained the best desorption rates; SDS is the best surfactant for loamy sand and silty clay soil and SDBS for silt loam soil. This study suggests that the concentration of soil organic matter in the soil is an irrelevant factor or, in any case, it has a lesser influence on the elimination of gasoline and diesel hydrocarbons than other parameters as the type of surfactant, the texture of the soil, and the size and chemical structure of hydrocarbons. Highlighting the significance of the different hydrocarbon compounds in the gasoline and diesel, the smallest hydrocarbons were desorbed in soil more than those of the longest range. Also, the results indicate that aromatic compounds are removed from soil slightly better than aliphatic hydrocarbons. The detailed study carried out on the different hydrocarbon groups that comprise of gasoline and diesel fuels provides valuable information on how to remove the most toxic and dangerous compounds present in these abundant fuels from soil and groundwater. The optimization of surfactant choice based on the characteristics of the polluted place might reduce remediation time, save a quantity of chemical reagents, and therefore make the remediation on a full scale more cost-effective.

## References

[CR1] Agency for Toxic Substances and Disease Registry (ATSDR) (1999). Toxicological profile for total petroleum hydrocarbons.

[CR2] Alaska Statement of Cooperation Working Group (2006) Hydrocarbon characterization for use in the hydrocarbon risk calculator and example characterizations of selected Alaskan fuels. Tech. Backgr. Doc. Recomm

[CR3] Atteia O, Jousse F, Cohen G, Höhener P (2017). Comparison of residual NAPL source removal techniques in 3D metric scale experiments. J. Contam. Hydrol..

[CR4] Baziar M, Mehrasebi MR, Assadi A, Fazli MM, Maroosi M, Rahimi F (2013). Efficiency of non-ionic surfactants - EDTA for treating TPH and heavy metals from contaminated soil. J. Environ. Heal. Sci. Eng..

[CR5] Brownawell BJ, Chen H, Zhang W, Westall JC (1997). Sorption of nonionic surfactants on sediment materials. Environ. Sci. Technol..

[CR6] Ceschia E, Harjani JR, Liang C, Ghoshouni Z, Andrea T, Brown RS, Jessop PG (2014). Switchable anionic surfactants for the remediation of oil-contaminated sand by soil washing. RSC Adv..

[CR7] Chattopadhyay P, Karthick RA (2017). Characterization and application of surfactant foams produced from ethanol-sodium lauryl sulfate-silica nanoparticle mixture for soil remediation. Macromol. Symp..

[CR8] Cheng M, Zeng G, Huang D, Yang C, Lai C, Zhang C, Liu Y (2018). Tween 80 surfactant-enhanced bioremediation: toward a solution to the soil contamination by hydrophobic organic compounds. Crit. Rev. Biotechnol..

[CR9] Chevalier LR (2003). Surfactant dissolution and mobilization of LNAPL contaminants in aquifers. Environ. Monit. Assess..

[CR10] Deshpande S, Shiau BJ, Wade D, Sabatini DA, Harwell JH (1999). Surfactant selection for enhancing ex situ soil washing. Water Res..

[CR11] Dhaka A, Chattopadhyay P (2021). A review on physical remediation techniques for treatment of marine oil spills. J. Environ. Manage..

[CR12] Duplâtre G, Ferreira Marques MF, da Graça Miguel M (1996). Size of sodium dodecyl sulphate micelles in aqueous NaCl solutions as studied by positron annihilation lifetime spectroscopy. J. Phys. Chem..

[CR13] Federle TW, Itrich NR (2006). Fate of free and linear alcohol-ethoxylate-derived fatty alcohols in activated sludge. Ecotoxicol. Environ. Saf..

[CR14] Fernández Linares LC, Rojas Avelizapa NG, Roldán Carrillo TG, Ramírez Islas ME, Zegarra Martínez HG, Uribe Hernández R, Reyes Ávila RJ, Flores Hernández D, Arce Ortega JM (2006). Manual de Técnicas de análisis de suelos aplicadas a la remediación de sitios contaminados.

[CR15] Gustav K, Jurgen R, Belanger S, Gamon K, Sedlak R (2011). Environmental properties and aquatic hazard assessment of anionic surfactants: physico-chemical , environmental fate and ecotoxicity properties. Ecotoxicol. Environ. Saf..

[CR16] Ishiguro M, Koopal LK (2016). Surfactant adsorption to soil components and soils. Adv. Colloid Interface Sci..

[CR17] Jousse F, Atteiaa O, Höhener P, Cohen G (2017). Removal of NAPL from columns by oxidation, sparging, surfactant and thermal treatment. Chemosphere.

[CR18] Karjiban RA, Basri M, Rahman MBA, Salleh AB (2012). Structural properties of nonionic Tween 80 micelle in water elucidated by molecular dynamics simulation. APCBEE Procedia.

[CR19] Karthick RA, Chattopadhyay P (2017). Remediation of diesel contaminated soil by Tween-20 foam stabilized by silica nanoparticles. Int. J. Chem. Eng. Appl.

[CR20] Karthick A, Chauhan M, Krzan M, Chattopadhyay P (2019). Potential of surfactant foam stabilized by ethylene glycol and allyl alcohol for the remediation of diesel contaminated soil. Environ. Technol. Innov..

[CR21] Karthick A, Roy B, Chattopadhyay P (2019). A review on the application of chemical surfactant and surfactant foam for remediation of petroleum oil contaminated soil. J. Environ. Manage..

[CR22] Karthick A, Roy B, Chattopadhyay P (2019). Comparison of zero-valent iron and iron oxide nanoparticle stabilized alkyl polyglucoside phosphate foams for remediation of diesel-contaminated soils. J. Environ. Manage..

[CR23] Khalladi R, Benhabiles O, Bentahar F, Moulai-Mostefa N (2009). Surfactant remediation of diesel fuel polluted soil. J. Hazard. Mater..

[CR24] Lake LW (1998). Enhanced oil recovery.

[CR25] Lee D, Cody RD, Kim D, Choi S (2002). Effect of soil texture on surfactant-based remediation of hydrophobic organic-contaminated soil. Environ. Int..

[CR26] Li G, Guo S, Hu J (2016). The influence of clay minerals and surfactants on hydrocarbon removal during the washing of petroleum-contaminated soil. Chem. Eng. J..

[CR27] Liu Z, Edwards DA, Luthy RG (1992). Sorption of non-ionic surfactants onto soil. Water Res..

[CR28] López J, Iturbe R, Torres LG (2004). Washing of soil contaminated with PAHs and heavy petroleum fractions using two anionic and one ionic surfactant: effect of salt addition. J. Environ. Sci. Heal. - Part A Toxic/Hazardous Subst. Environ. Eng..

[CR29] Mao X, Jiang R, Xiao W, Yu J (2015). Use of surfactants for the remediation of contaminated soils: a review. J. Hazard. Mater..

[CR30] Muherei MA, Junin R, Bin Merdhah AB (2009). Adsorption of sodium dodecyl sulfate, Triton X100 and their mixtures to shale and sandstone: a comparative study. J. Pet. Sci. Eng..

[CR31] Ou Z, Yediler A, He Y, Jia L, Kettrup A, Sun T (1996). Adsorption of linear alkylbenzene sulfonate (LAS) on soils. Chemosphere.

[CR32] Palazzesi F, Calvaresi M, Zerbetto F (2011). A molecular dynamics investigation of structure and dynamics of SDS and SDBS micelles. Soft Matter.

[CR33] Panagos P, Van Liedekerke M, Yigini Y, Montanarella L (2013). Contaminated sites in Europe: review of the current situation based on data collected through a European network. J. Environ. Public Health..

[CR34] Paria S (2008). Surfactant-enhanced remediation of organic contaminated soil and water. Adv. Colloid Interface Sci..

[CR35] Paria S, Yuet PK (2006). Effects of chain length and electrolyte on the adsorption of n-alkylpyridinium bromide surfactants at sand-water interfaces. Ind. Eng. Chem. Res..

[CR36] Peng S, Wu W, Chen J (2011). Removal of PAHs with surfactant-enhanced soil washing : influencing factors and removal effectiveness. Chemosphere.

[CR37] Pennell KD, Abriola LM, Weber WJ (1993). Surfactant-enhanced solubilization of residual dodecane in soil columns. 1. Experimental investigation. Environ. Sci. Technol..

[CR38] Podoll RT, Irwin KC, Brendlinger S (1987). Sorption of water-soluble oligomers on sediments. Environ. Sci. Technol..

[CR39] Preu H, Zradba A, Rast S, Kunz W, Hardyc EH, Zeidlerc MD (1999). Small angle neutron scatterin of D2O-Brij 35 and D2O-alcohol-Brij 35 solutions and their modelling using the Percus-Yevick integral equation. Phys. Chem. Chem. Phys..

[CR40] Rodríguez-Cruz MS, Sánchez-Martín MJ, Sánchez-Camazano M (2005). A comparative study of adsorption of an anionic and a non-ionic surfactant by soils based on physicochemical and mineralogical properties of soils. Chemosphere.

[CR41] Shen Y (2000). Sorption of non-ionic surfactants to soil : the role of soil mineral composition. Chemosphere.

[CR42] Tabor CF, Barber LB (1996). Fate of linear alkylbenzene sulfonate in the Mississippi River. Environ. Sci. Technol..

[CR43] The Risk Assessment Information System (2015) RAIS Database [WWW Document]. URL https://rais.ornl.gov/ (accessed 11.15.20)

[CR44] The Royal Netherlands Standardization Institute (NEN) (1992) Soil determination of organic matter content in soil as loss-on-ignition. NEN 5754

[CR45] The Royal Netherlands Standardization Institute (NEN) (2018) Soil determination of clay content and particle size distribution in soil and sediment by sieve and pipet. NEN 5753

[CR46] The Soap and Detergent Association (SDA) (1991). Environmental and human safety of major surfactants. Volume I. Anionic surfactants. Part 1. Linear alkylbenzene sulfonates.

[CR47] The Soap and Detergent Association (SDA) (1994). Environmental and human safety of mayor surfactants. Volume II: nonionic surfactants. Alcohol ethoxylates and alkylphenol ethoxylates.

[CR48] Tsai TT, Kao CM (2009). Treatment of petroleum-hydrocarbon contaminated soils using hydrogen peroxide oxidation catalyzed by waste basic oxygen furnace slag. J. Hazard. Mater..

[CR49] United States Environmental Protection Agency USEPA (1994) Method 3541 1–10

[CR50] United States Environmental Protection Agency USEPA (1996a) 8260b Method 1–86

[CR51] United States Environmental Protection Agency USEPA (1996b) Method 8015B 1–28

[CR52] United States Environmental Protection Agency USEPA, (1996c) Method 3540C 1–8

[CR53] United States Environmental Protection Agency USEPA (2013). Superfund Remedy Selection Report, 14th Editi. ed.

[CR54] United States Environmental Protection Agency USEPA, 2014. 5021a Method 1–31.

[CR55] Urum K, Grigson S, Pekdemir T, McMenamy S (2006). A comparison of the efficiency of different surfactants for removal of crude oil from contaminated soils. Chemosphere.

[CR56] Ussawarujikulchai A (2008). Synergistic effects of organic contaminants and soil organic matter on the soil-water partitioning and effectiveness of a nonionic surfactant (Triton X-100). Bioremediat. J..

[CR57] Vreysen S, Maes A (2005). Remediation of a diesel contaminated, sandy-loam soil using low concentrated surfactant solutions. J. Soils Sediments.

[CR58] Yan G, Ma W, Chen C, Wang Q, Guo S, Ma J (2016). Combinations of surfactant flushing and bioremediation for removing fuel hydrocarbons from contaminated soils. Clean - Soil, Air, Water.

[CR59] Yang K, Zhu L, Xing B (2007). Sorption of sodium dodecylbenzene sulfonate by montmorillonite. Environ. Pollut..

[CR60] Zhao B, Zhu L, Gao Y (2005). A novel solubilization of phenanthrene using Winsor I microemulsion-based sodium castor oil sulfate. J. Hazard. Mater..

[CR61] Zheng Z, Obbard JP (2002). Evaluation of an elevated non-ionic surfactant critical micelle concentration in a soil/aqueous system. Water Res..

[CR62] Zhou W, Zhu L (2005). Distribution of polycyclic aromatic hydrocarbons in soil–water system containing a nonionic surfactant. Chemosphere.

[CR63] Zhou W, Zhu L (2007). Efficiency of surfactant-enhanced desorption for contaminated soils depending on the component characteristics of soil-surfactant–PAHs system. Environ. Pollut..

[CR64] Zhu L, Yang K, Lou B, Yuan B (2003). A multi-component statistic analysis for the influence of sediment/soil composition on the sorption of a nonionic surfactant (Triton X-100) onto natural sediments/soils. Water Res..

[CR65] Zhu K, Hart W, Yang J (2005). Remediation of petroleum-contaminated loess soil by surfactant-enhanced flushing technique. J. Environ. Sci. Heal..

